# Be cautious with the semilunar fold! Endoscopic perforation after cap-suction pseudopolyp formation for underwater en bloc resection of a big cecal lesion

**DOI:** 10.1055/a-2550-8494

**Published:** 2025-05-28

**Authors:** Harold Benites-Goñi, Paulo Bardalez, Luis Marin, Bryan Medina, Jairo Asencios, Hugo Uchima

**Affiliations:** 1 Endoscopy Service, Gastroenterology Department, Hospital Nacional Edgardo Rebagliati Martins, Lima, Peru; 2 Vicerrectorado de Investigación, Universidad San Ignacio de Loyola, Lima, Peru; 3 Endoscopy Unit, Teknon Medical Center, Barcelona, Spain; 4 Endoscopy Unit, Gastroenterology Department, Hospital Universitari Germans Trias i Pujol, Badalona, Spain

Use of underwater endoscopic mucosal resection (UEMR) has spread worldwide since its first description in 2012 by Binmoeller 1 . In a recent randomized controlled trial, UEMR was found to be superior to EMR, with lower recurrence rates for lesions sized 20–30 mm as well as being faster and easier, but with similar safety and overall effectiveness 2 .

Cap-suction pseudopolyp formation during UEMR (CAP-UEMR) is a safe and effective modified underwater technique that could be helpful in some complex situations 3 . This technique is based on creating a pseudopolyp by suctioning the lesion using a conical cap while submerged underwater to allow adequate capture with the snare for resection. Here, we report an infrequent case in which full-thickness resection appeared after performing CAP-UEMR ( Video 1 ).

Endoscopic perforation after cap-suction pseudopolyp formation for underwater en bloc resection of a big cecal lesion.Video 1

A 61-year-old woman was referred to our hospital for resection of a 35-mm 0-IIa+IIc cecal lesion located over a fold ( Fig. 1 ). Before resection, the lesion was classified as a nongranular pseudodepressed JNET 2B lesion ( Fig. 2 ).

**Fig. 1:**
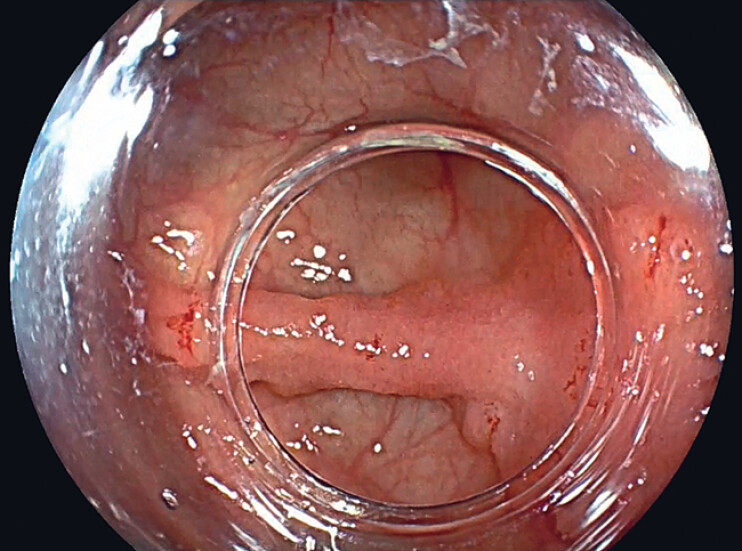
A 35-mm 0-IIa+IIc cecal lesion located over a fold.

**Fig. 2:**
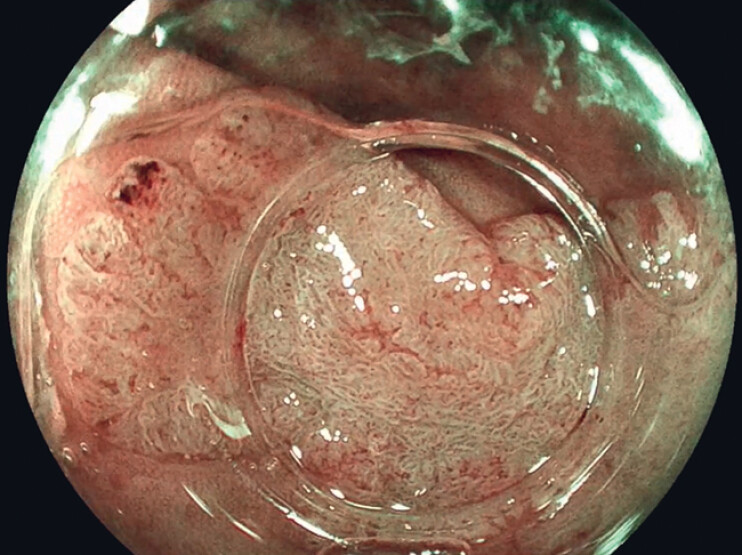
Virtual chromoendoscopy with blue-light imaging (Fujifilm Co., Tokyo, Japan).

When attempting to perform a classic UEMR procedure, difficulty was encountered in capturing the lesion, so we re-entered with a conical cap to apply CAP-UEMR, aiming for en bloc resection of the lesion. Cap aspiration was applied six times, with slight traction of the endoscope during aspiration to facilitate the creation of the pseudopolyp. At the end of the resection, we found that a full-thickness resection had occurred ( Fig. 3 ), so the defect had to be closed with clips ( Fig. 4 ).

**Fig. 3:**
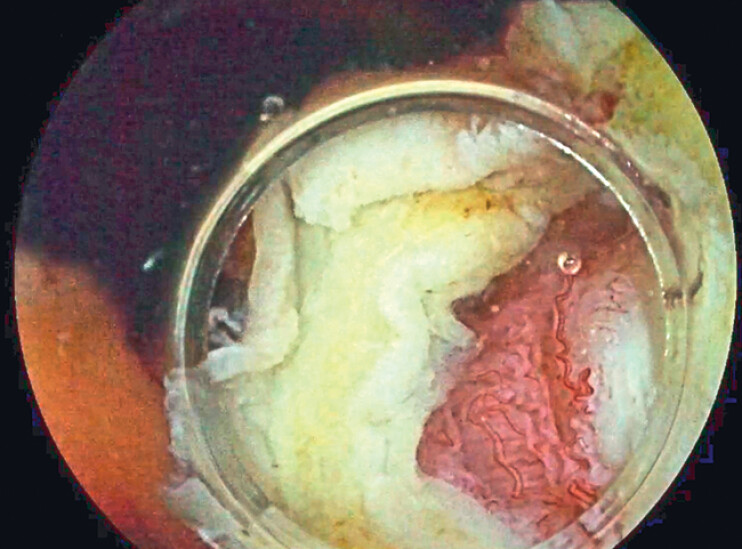
Full-thickness resection.

**Fig. 4:**
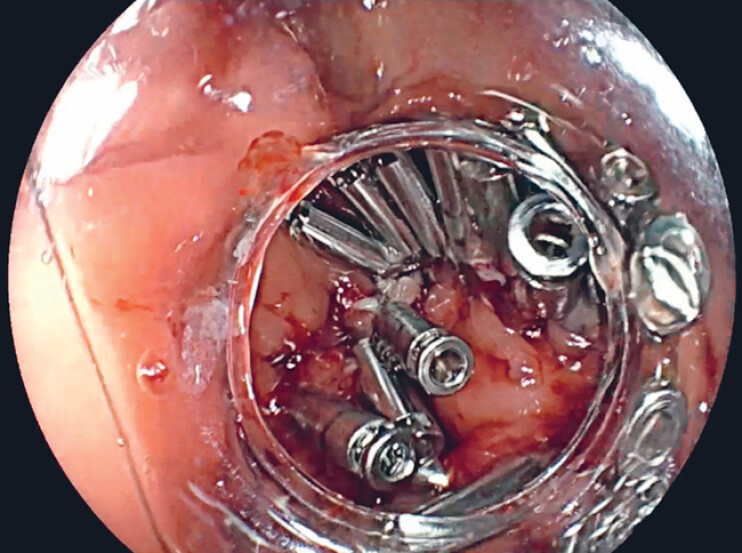
Defect closure with clips.

Antibiotics were started at the time of closure, and the patient was discharged without complications 7 days later. Final histology was tubular adenoma with focal high grade dysplasia and transition into a well-differentiated invasive adenocarcinoma with infiltration of the submucosa (pT1 (sm1) L0 V0 R0 G1).

Perforation risk after pseudopolyp formation during CAP-UEMR should be as low as during UEMR without cap suction pseudopolyp formation 2 . We think that caution must be taken when performing conical cap aspiration over semilunar folds, especially in pseudodepressed lesions, as there may be a greater risk of perforation, as indicated in previous reports of endoscopic resection, especially in the cecum where the muscle wall is thinner 4 5 . An excessive number of aspirations could be another risk factor, especially if they are performed over a semilunar fold, as the muscularis propria could be aspirated into the cap.

Endoscopy_UCTN_Code_CPL_1AJ_2AD_3AC

Citation Format

Endoscopy 2025; 57: E124–E125. DOI: 10.1055/a-2515-4089

## Notes

Endoscopy E-Videos https://eref.thieme.de/e-videos
*E-Videos* is an open access online section of the journal *Endoscopy* , reporting on interesting cases and new techniques in gastroenterological endoscopy. All papers include a high-quality video and are published with a Creative Commons CC-BY license. Endoscopy E-Videos qualify for HINARI discounts and waivers and eligibility is automatically checked during the submission process. We grant 100% waivers to articles whose corresponding authors are based in Group A countries and 50% waivers to those who are based in Group B countries as classified by Research4Life (see: https://www.research4life.org/access/eligibility/ ). This section has its own submission website at https://mc.manuscriptcentral.com/e-videos .
